# Can Rotational Grouping Be Determined by the Initial Conditions?

**DOI:** 10.1177/2041669517748338

**Published:** 2018-01-22

**Authors:** Allan C. Dobbins, Jon K. Grossmann

**Affiliations:** Department of Biomedical Engineering, Vision Science Research Center, 9968 University of Alabama at Birmingham, AL, USA

**Keywords:** kinetic depth effect, structure from motion, bistable perception, perceptual grouping, transparency, initial conditions

## Abstract

Objects rotating in depth with an ambiguous rotation direction frequently appear to rotate together. Corotation is especially strong when the objects are interpretable as having a shared axis. We manipulated the initial conditions of the experiment by having pairs of objects initially appear to be unambiguous, and then make either a sudden or gradual transition to ambiguous spin. We find that in neither case do coaxial counter-rotating objects persist in being perceived as counter-rotating. This implies that the perceptual constraint that favors coaxial corotation overrides the initial perceptual state of the objects.

## Introduction

**Figure 1.**
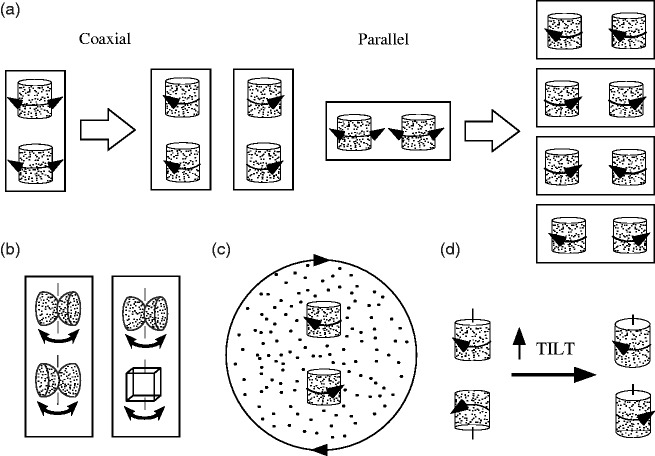
Rotational grouping of transparent kinetic dot objects. (a) Coaxial objects tend to be perceived as rotating together (∼80%–95% of the time), whereas for parallel objects, shared rotation is only slightly more common than opposite rotation. (b) Coaxial corotation does not depend on the objects having “good continuation” (radar dishes are 90° out of phase) or the same form. (c) The objects can be biased in a variety of ways to break coaxial coupling, for example, by adding suitable binocular disparity, or here by superimposing a rotating planar flow field that induces a bottom-up bias and leads to perceived counter-rotation (see Demo 1). (d) If the objects are objectively counter-rotating, tilting the virtual camera at some point leads to a perceptual transition from common spin to common axis interpretation (see Demo 2).

Perceptual grouping has been a subject of fascination from the time of the Gestalt psychologists beginning about a century ago (e.g., Koffka, 1935; Köhler, 1920; Wertheimer, 1923). However, despite the appeal of simplification, it is far from clear that grouping processes can be unified in a single framework (Zucker, 1987). For example, one sense of grouping is the incorporation of one kind of element into a different class of element, such as tangents into curves or texture elements into surfaces. A quite different sense of grouping occurs when distinct entities, which share an ambiguous property, all acquire the same value of that property. Consider the case of objects ambiguously rotating in depth. Presented with an array of transparent kinetic dot objects with a shared axis of rotation in the plane of the screen, there is a strong tendency to see all the objects spin in the same way, and when they undergo a perceptual switch to do so synchronously or nearly so (see Dobbins & Grossmann, 2010, for a counterexample). Gillam (1972) showed that parallel line segments ambiguously rotating in depth about a shared vertical axis tend to be perceived as rotating the same way, although the effect diminished to chance with increasing angular difference between the lines. In other experiments by Gillam and coworkers, the probability of line grouping depended in a complex way on the parameters (see Discussion section). Eby, Loomis, and Solomon (1989) demonstrated this same tendency for coaxial corotation with transparent, kinetic dot objects. In contrast, if two rotating objects are displaced parallel to the axis of rotation instead, they are often seen to rotate independently (Long & Toppino, 1981). This is illustrated in Figure 1(a)—coaxial transparent cylinders appear to rotate together about 90% of the time, while for parallel cylinders, perceived corotation only slightly exceeds counter-rotation (50%–65% corotation depending on parametric details; Dobbins, Grossmann, & Smith, 1998), unless they are touching, in which case the opposite rotation (“frictional” or “gear meshing”) percept is more common (Gilroy & Blake, 2004). It is not essential that the coaxial objects be interpretable as part of a single object or exhibit good continuation as occurs with identical cylinders. With objects composed of back-to-back hemispheres (“radar dishes”) having a 90° rotational phase difference between the objects, the motion flow fields and bounding contours of the coaxial objects change asynchronously, and yet there is still strong rotational coupling (Figure 1(b)). This remains true even with objects of different shape (Figure 1(b), Grossmann & Dobbins, 2003). On the other hand, it is possible to decouple coaxial objects in a variety of ways. Figure 1(c) is inspired by Sereno and Sereno (1999) who showed that a transparent kinetic dot object appears to have its front face rotate opposite to the direction of a parallel planar dot field. By using a rotating dot field, the local bias is opposite for the two objects and they appear to counter-rotate (see Demo 1). Any manipulation that increases the signal of one direction of dot motion with respect to the other increases its likelihood of representing the front face of the object and thus determining the rotation direction.

In the usual situation, the axis of rotation is in the plane of the viewing screen. In a way, this is a degenerate case or singular viewpoint. For example, if the objects are in fact counter-rotating in the virtual world, then changing the virtual viewing position (or equivalently, tilting the objects) decouples the spin axis and spin direction. In earlier experiments, we found that at small tilts, common spin dominated, but that with increasing tilt, the alternative percept (shared common axis with opposite spin) became more common (Figure 1(d), see Demo 2). In this situation, there are two alternative perceptual models or groupings. Coaxial grouping can be modified by either bottom up biases, or plausibly, by top down model-based constraints. There is one further factor that is worth mentioning. In binocular rivalry and bistable perception, transient biases are independent of long-term biases. For example, for one of the authors, the first impression of objects such as those in Figure 1(a) and (b) is of rightward rotation. Yet, he has no long-term or steady-state bias for rotation direction. A similar dissociation has been reported in binocular rivalry (Carter & Cavanagh, 2007). This led us to wonder if by precisely controlling the initial conditions of the display, we could control the probability of perceptual grouping into any particular perceptual state. Therefore, we undertook the two experiments reported here in which a pair of rotating dot objects are displayed so as to be initially unambiguous in their sense of rotation and then undergo either an abrupt or smooth transition to ambiguous rotation. This is possible based on two observations: (a) if the dots in a kinetic dot object move in only one direction, this is almost invariably seen as the front surface of an opaque object; (b) if the dots in a kinetic dot object move in two opposite directions and the dots moving in one direction have higher contrast, these more energetic dots are seen as the front surface (Grossmann & Dobbins, 2003), first shown with wire frame cubes (Schwartz & Sperling, 1983). In the first experiment, the kinetic dot objects initially are opaque and then abruptly become transparent. The sudden appearance of the oppositely directed dots tends to cause an immediate perceptual reversal—the newly visible dots becoming the front surface. In the second experiment, initially opaque objects are joined by oppositely directed dots that ramp up to be equal in luminance to the initially visible dots. In each case, the question is, if the objects are initially counter-rotating, can this normally unfavored state persist when the evidence for the two alternatives becomes balanced?

## Methods

### Apparatus

Experiments were run on a Silicon Graphics Indigo 2 computer running custom software developed in the laboratory that employed the Open Inventor® and Open GL® libraries and the Guile scripting language (the GNU Project). The video monitor was a 20” Sony CRT (1280 × 1024 @ 76 Hz). Observers had their head centered via a chin rest and curved forehead restraint, adjusted so that eye level was at mid-screen. The display was viewed from a distance of 57 cm in a room with dim lighting to minimize screen reflections.

### Stimuli

Dot-covered cylinders (axial length: 3°, radius: 1.5°) were orthographically projected and rotated in depth at 20 r/min. Cylinders were centered at ± 3° displaced along the x or y axes (separated by one object diameter) so that the rotational axes were coaxial (four same spin and four opposite spin conditions) or parallel (four same spin and four opposite spin conditions). Each cylinder was covered by 100 randomly positioned dots (size: 2 × 2 pixels) of high contrast on a dark background (dots: 85 cd/m^2^; background: 4.8 cd/m^2^). Dot density was low enough that the bounding contours of cylinders were not clearly demarcated by dot position, and the dot size (∼3 arc min) was small enough so as to appear as dots rather than square texture objects.

### Subjects

Both authors and four naïve observers participated in the first experiment and one author and four naïve observers participated in the second experiment. All subjects had normal acuity. In addition, preliminary evaluation established that the observer could see an ambiguously rotating object in both interpretations and without a strong bias for one of the interpretations. This was determined by having one of the investigators sit with the candidate observer and having them verbally report each perceptual switch of a single rotating ambiguous kinetic dot object. Following this initial screening, participants were instructed how to do the experiment. This included going through the conditions in order from 1 to 16, pointing out the icons on the keyboard for that trial, having the observer preposition their hands, and then hitting the space bar to initiate the trial. Thus familiarized, each observer then underwent a practice session that involved running the full-length experiment. At the end of this practice run, one of the investigators entered the room, sat with the observer, and asked questions about the experience (Could they clearly see both objects throughout the trial? Did they have trouble making appropriate key responses? Did they have any observations to share?) Data from this session were examined by the investigators immediately after the run but not saved. On a following day, the participants ran the experiment again and the data obtained from that session were saved, analyzed, and are reported here. Experimental protocols were approved by the UAB institutional review board for research with human subjects.

### Experimental Design

In both experiments, a message box appeared on the screen to inform the experimenter of the type of trial (different trial types involved the use of different keys) to allow prepositioning of fingers. With fingers in place, a press of the Space Bar initiated the central fixation stimulus and the display of the two cylinder stimuli. In all trials, the observer used four keys (two per hand) to report the perceived rotational state of each object. The key positions in the different trials mimicked the spatial configuration of the objects on the screen, for example, in a vertical axis coaxial trial, two adjacent keys in the top row were labeled with left and right arrows for the upper object, and two adjacent lower row keys directly below the designated upper row keys were labeled with left and right arrows for the lower object. Because of the complexity of the task, the practice session the day before the experiment enabled the observers to gain fluency in the task. The experiment was self-paced with observers able to take breaks and look around between trials when desired.

Trials were 20 s in duration. There were 16 conditions to generate all combinations of coaxial and parallel configurations, horizontal and vertical rotational axes, and co- and counter-rotation in all positions. A block of trials consisted of each of the 16 conditions occurring once in random order, and there were 6 blocks of trials in Experiment 1, and 6, 8, or 10 blocks in Experiment 2 (two observers had 6, two observers had 8, and one observer had 10 across two sessions). Six versus eight blocks was an accidental change in the experimental script, while the observer (one of the authors) who viewed 10 blocks was a result of running two experimental sessions on separate days with additional control conditions.

### Experiment 1—Step

Ten seconds into each trial, the dots on the rear surface of the cylinders made a sudden transition from invisibility to visibility. Initially, cylinders might be corotating or counter-rotating, but once the rear surface dots appeared, there was equal evidence for both interpretations of rotation direction (20 s total).

### Experiment 2—Ramp

Five seconds into each trial, the rear surface dots (initially invisible) began a 10-s linear ramp up of luminance, equaling the front surface dots 15 s into the trial. The trial continued for an additional 5 s at equal luminance (20 s total).

### Videos

Included are several videos to illustrate the different trial types with self-explanatory file names that replicate each of the four types of experimental trials in the two experiments. Note that these QuickTime videos are generated with different software run on a different computer (Mac OS X 10.11 El Capitan), and although some effort was made, they are not precisely the same as the original real-time animations in the corresponding experiment. However, they are close enough to provide the viewer a better sense of the experimental stimuli than is provided by text description alone (for guide to movie files, see online Table 1).

## Results

### Guide to Figures

The figures have a consistent color code. Dark blue represents the time before the first response at trial onset and pale blue (State 1) represents the period of the initial unambiguous percept. The dark brown color (State 2) represents its opposite—the rebound state when each cylinder is perceived as spinning opposite to its initial state. Therefore, for same-spin initial configurations, brown represents same-spin but in the opposite direction to the initial state, whereas for opposite-spin initial configurations, brown represents the complementary opposite-spin configuration. In all cases, therefore, brown represents the perceptual state representing the switch to the state in which both objects spin oppositely to the initial state. On the other hand, the interpretation of the green (State 3) and mustard states (State 4) varies—they are always the states that are neither the initial state or opposite-to-initial state, but they correspond to counter-rotation in the initially corotating trials (top two panels) and to corotation in the initially counter-rotating trials (bottom two panels) of Figures 3 and 5.

**Figure 2.**
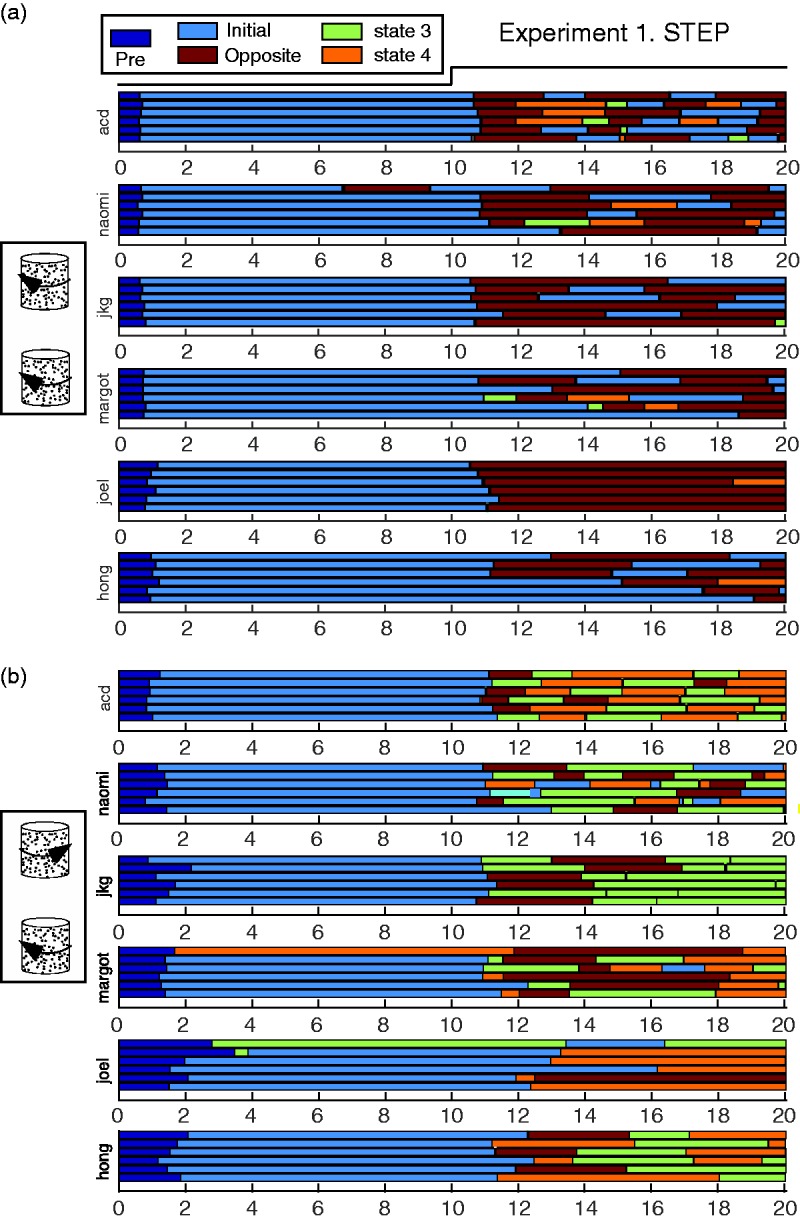
All individual data for two experimental conditions. (a) Raw data for the condition in Experiment 1 in which coaxial objects initially rotate to the left. Despite the difference in rate of perceptual switching among observers, in almost every trial, the first response after the opaque-transparent transition at the 10-s mark is to report seeing both cylinders spinning to the right (Opposite). In 33 of the 36 trials, at the end of the trial, the observers are reporting one of the corotation percepts (initial or opposite). (b) Raw data for one of the vertical coaxial, counter-rotation conditions. The opposite-to-initial percept is not necessarily the first following the stimulus transition and does not dominate. Rather, the corotation states (3 and 4) appear to be most common.

**Figure 3.**
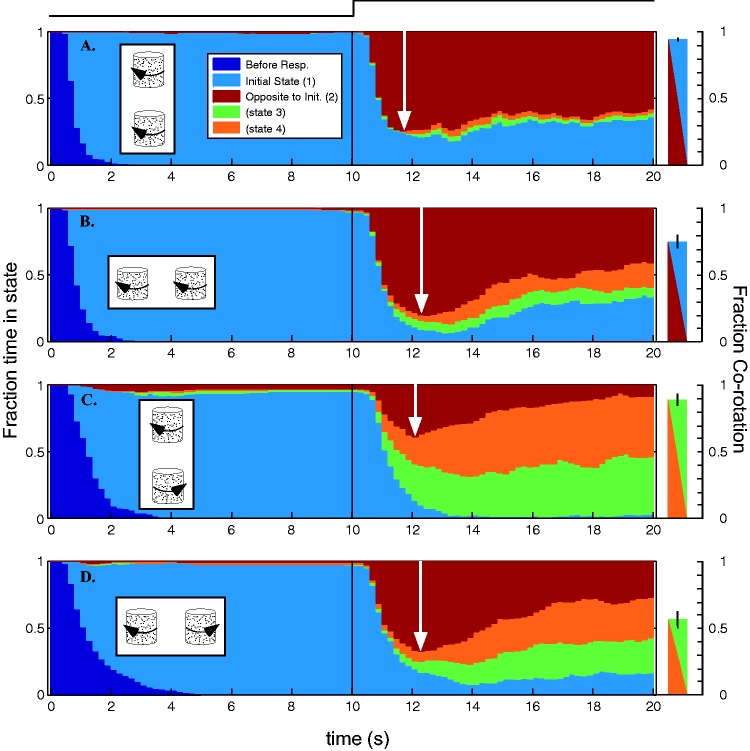
Temporal dynamics of step transition to transparency. A pair of kinetic dot cylinders rotates unambiguously for 10 s, then instantly transitions to being ambiguous. The graph represents the time evolution of the four perceptual states summed over all subjects. Initial configuration: (a) Coaxial corotation; (b) Parallel corotation; (c) Coaxial counter-rotation; (d) Parallel counter-rotation. Left column: In the first half of the trial, observers report seeing the opaque objects veridically essentially all the time (pale blue). Brown represents the local rebound effect since both objects switch. At 1.5 to 2.5 s posttransition, observers are most likely to be in this perceptual state. The white arrow represents the fraction of time in State 2 (brown) at ∼12 s. Note that for coaxial counter-rotation (c) the magnitude of the local rebound effect (white arrow) is substantially lower at 40%. The probability of perceiving the local aftereffect (brown) diminishes from the peak, but how much depends on whether it is consistent with corotation (a, b) or counter-rotation (c, d). In the latter conditions, corotation (mustard and green) increase as the local aftereffect diminishes. Right column: The bar plots illustrate the fraction of time the observers perceive corotation at the end of the trial: ∼90% corotation in the coaxial conditions and ∼55% to 75% in the parallel conditions. The difference reflects the strong tendency toward perceiving coaxial corotation. The colors blue/brown or green/mustard represents the two perceptual states on the left that are summed to represent corotation in each condition (Error bars: *SEM*).

### Response Latency

Figure 2 shows all of the individual trial data for Conditions 1 (vertical coaxial—initial rotation left) and 3 (vertical coaxial—initial top right, bottom left). Dark blue represents the time before the first response at trial onset and pale blue (State 1) represents the period of the initial unambiguous percept. Individual observers have mean latencies ranging from ∼0.6 s to ∼1 s. The distribution of initial response latencies for all four trials types collapsed across observers can be seen at the left of Figure 3. Note that the median latency is higher and the distribution broader in the counter-rotation conditions (Figure 3(c) and (d)). 

After the transition from opaque to transparent at the 10 s mark, observers make a perceptual switch with latencies slightly longer than the initial response, but some observers exhibit much greater variance with a mix of short and long latency responses (Figure 2). The median latency range seen in the initial response is at best a rough guide to the latencies expected for later responses. With this point in mind, perceptual transitions are probably at least a second earlier than their accompanying reports. The implication is that the perceptual transitions reported within 1 to 2 s of the step transition at mid-trial in Figures 2 and 3 probably occurred almost immediately after the step transition.

### Experiment 1

**Figure 4.**
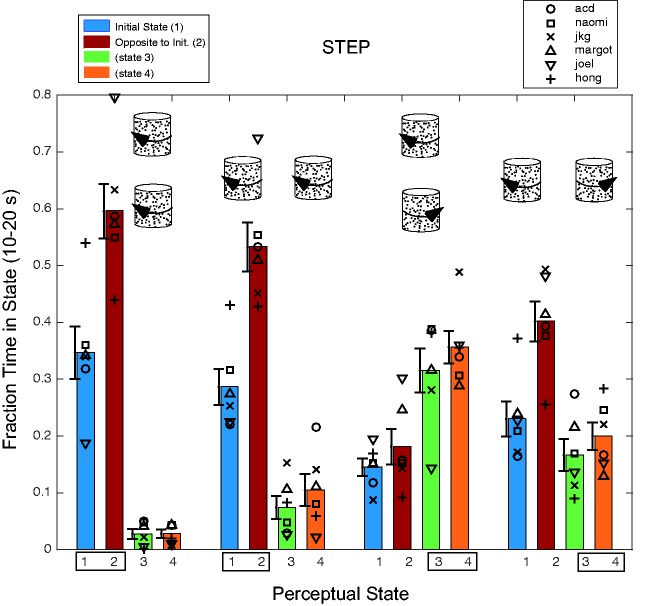
Time in each state during second phase of trial during step experiment. Each group of four bars represents the merging of four different experimental conditions (horizontal and vertical axes and both senses of spin). The icons above a group of bars represent one object or spin configuration in the group. The bars show the fraction of time spent in each of the four perceptual states for the last 10 s of the trial when the objects are transparent. The small rectangles at the bottom enclosing either States 1 and 2 or States 3 and 4 represent the same-spin perceptual states. For all but the coaxial counter-rotation conditions, State 2—the local rebound effect—in which each object is seen to rotate opposite to the initial unambiguous percept—is the most common. Error bars (*SEM*) are displaced to the left so as to not obscure individual data markers.

Figures 3 and 4 shows the results of the step experiment in which the cylinders instantly transition from opaque to transparent halfway through each trial.

**Figure 5.**
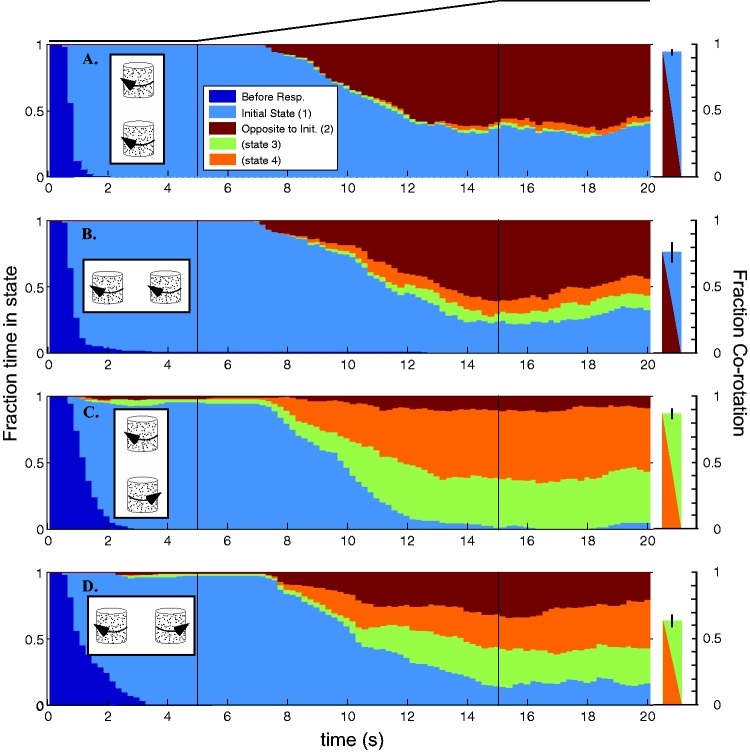
Temporal dynamics during ramp transition to transparency. For the first 5 s, an object’s visible dots move in only one direction and are interpreted as the front surface. Beginning at 5 s, back surface dots begin a 10-s linear ramp up in luminance. At 15 s, they are equal in luminance to the front surface dots and so each object is balanced in energy for the trial’s final 5 s. All conventions are the same as in Figure 3. The principal difference is that there is no clear peak in the local rebound effect (State 2—brown), instead there is a slow increase in probability of a transition beginning within about 2 s of the ramp onset. The distribution of perceptual states is very similar in the final seconds of corresponding conditions in both experiments, and this is summarized in the right column which depicts the proportion of time seeing corotation at the end of trial.

Figure 3 illustrates the time evolution of the different percepts summed over all observers. One can think of this as summing all of the individual data (e.g., as shown for Conditions 1 and 3 in Figure 2) in small time bins to create a stacked histogram. At any given time, the sum of the different states must add to one. To aid interpretation, note that the opposite-to-initial or rebound state (brown) is always on the top of the stack and so its magnitude can be measured down from the top of the plot. There are two main points: (a) there is a rebound effect that occurs very rapidly (peak at ∼12 s—indicated by white arrows) in all conditions and (b) the coaxial counter-rotation rebound (Figure 1(c)) is much smaller than in the other conditions (∼ 40% peak at 12 s, diminishing to less than 10% by the end of the trial) and corotation predominates within 3 or 4 s. In other words, it is not possible to sustain the perception of coaxial counter-rotation even when biased into that state by the rebound effect induced by initial adaptation plus step change.

For three of the four classes of conditions, the opposite-to-initial or rebound state quickly becomes dominant (A: 75%, B: 80%, D: 67% at ∼12 s (Figure 3, white arrows)). However, the time evolution of the perception then varies in the different conditions. For example, in A (initial-coaxial-corotation), there is a slow recovery of the initial coaxial state. In other words, both cylinders tend to switch in perceived rotation together with little single object switching, and hence very little perceived counter-rotation. A similar result is seen in B (initial-parallel-corotation), with the gradual recovery of the initial perceived rotation state accompanied by some single object switching, leading to substantially more perceived counter-rotation (green and mustard). The opposite rotation conditions are similar to each other in the sense that the recovery of the initial percept (pale blue) is absent (C) or weak (D), and the two same-rotation percepts (mustard and green) grow rapidly as the rebound effect diminishes. A difference between the initial corotation (A and B) and counter-rotation (C and D) conditions is that in the latter, one can estimate a time constant (1/e) of recovery from the rebound effect (C: ∼4 s, D: ∼5.5 s), whereas in the initial corotation conditions, the local rebound percept never declines to 1/e of its peak value and remains the most probable state throughout the trial.

The bars at the right side of Figure 3 collapse the four perceptual states to show the fraction of corotation at the end of the trial. The bars are color-coded to illustrate the two states that compose corotation in each condition. The coaxial conditions (Figure 3(a) and (c)) have the greatest corotation at the end of the trial (A—initial corotation: 94%, C—initial counter-rotation: 88%) while the parallel conditions have rather less (B—initial corotation: 74%, D—initial counter-rotation: 56%). In the coaxial conditions, at the end of trial, observers perceived corotation in 265 out of 288 or 92% of trials. The end-of-trial difference in corotation between A and C and between B and D represents the residual of the rebound effect at 10 s posttransition. Figure 7 summarizes end-of-trial corotation data for both experiments. To summarize the main point, there is a strong coaxial corotation constraint and the experimental manipulation failed to sustain coaxial counter-rotation beyond a transient rebound effect. 

Figure 4 shows the fraction of time spent in each of the four perceptual states in the last 10 s of the trial when rotation is ambiguous. In the initially corotating conditions, observers spend the majority of the 10 s perceiving corotation opposite to the initial state, followed by the initial corotation percept, with very little time perceiving counter-rotation. The pattern is very different in the initially counter-rotating conditions. For the initial coaxial counter-rotation case, the corotation percepts are more frequent that either the initial or rebound percepts, while in the parallel counter-rotation case, the rebound state is the most prevalent. The most salient feature of the data is the discrepant coaxial counter-rotation conditions—in which only 18% of time is spent in the rebound state and only 32% in States 1 and 2 combined—much less than in the three other object configurations.

Figures 3 and 4 show that in the coaxial counter-rotation conditions, the peak rebound effect is smaller than the other conditions and much less time is spent in that state. If the rebound were primarily attributable to a local, classical motion aftereffect, one would not expect the early peak aftereffects to vary so dramatically.

### Experiment 2

**Figure 6.**
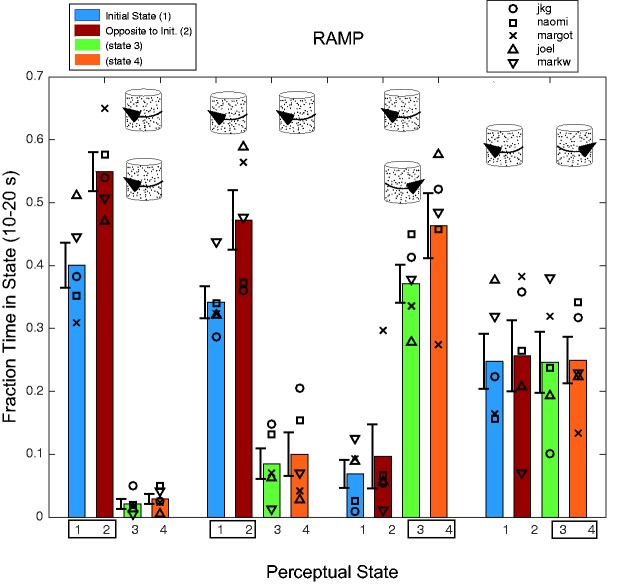
Time in each state during second phase of trial during ramp experiment. As in Figure 4, the bars depict the fractional time in each perceptual state for the last 10 s of the trial. For the coaxial and parallel corotation trials (two left groups), observers spend more time perceiving the corotation opposite to the initial state. In the coaxial counter-rotation condition, the most frequently perceived states are corotation (States 3 and 4) with very little time spent in the opposite-to-initial state (State 2).

**Figure 7.**
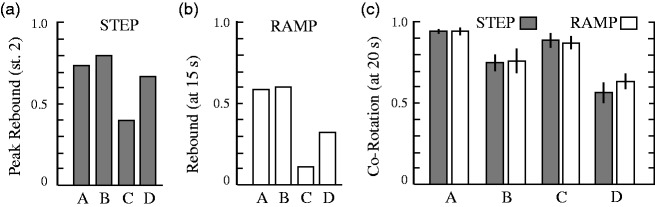
Summary of the two experiments. Peak perception of the rebound (opposite-to-initial) state in (a) step and (b) ramp experiments ((a) to (d) under bars refer to corresponding classes of conditions in Figures 3 to 6). The pattern is quite similar in the two experiments with a stronger peak aftereffect (by 10% to 20%) in the step experiment. In both experiments, the coaxial counter-rotation conditions exhibit a notably smaller peak aftereffect. (c) A comparison that demonstrates the similarity of the fraction of corotation at the end of trial in step and ramp experiments. Corotation is greater in (a) than (c), and greater in (b) than (d) at trial’s end, indicating that initial corotation (and its local rebound corotation) have a persistent advantage compared with the initial counter-rotation conditions, in which corotation and the local rebound effect are incompatible.

Figures 5 and 6 illustrate the results of the ramp experiment. Although the structure of Experiment 2 is different with a slow transition from opaque to transparent, analogously with Figure 1, Figure 6 is obtained from the second 10-s epoch of each trial. The data could have been analyzed differently, for example, examining the post-ramp 5 s of each trial, but Figure 5 shows that the observers begin making perceptual transitions within a few seconds of the ramp onset. Therefore, for ease of comparison, Figure 6 uses the last 10 s. Again, Figure 5 shows the time evolution of the percepts in the different conditions and Figure 7 summarizes and compares the step and ramp experiments.

Figure 5 shows that in all conditions, perceptual switches become noticeable within 2 to 3 s of the beginning of the increase in dot luminance. Unlike the step experiment, there is not a clearly demarcated transient peak and decay in the opposite-to-adapt (rebound) perceptual state (brown). In the four conditions that comprise Panel C, there are two main points to note: (a) the initial counter-rotating perceptual state gradually diminishes to become negligible as rear face dot luminance ramps up and (b) the opposite-to-initial rebound state (perceptual counter-rotation) represents 10% or less of the percepts throughout the trial. Therefore, as in the first experiment, perception of coaxial counter-rotation becomes rare. As in Figure 3, the right column bars depict fraction of corotation at trial’s end.

Figure 6 shows that for the initial corotation conditions, more time is spent in the rebound percept. In the initial coaxial counter-rotation conditions, very little time is spent in the initial or rebound states—corotation predominates. The results are qualitatively very similar to the step experiment (Figure 4) with two exceptions: (a) in the initial coaxial counter-rotation conditions, perceived corotation is more predominant in the ramp experiment and (b) in the initial parallel counter-rotation condition, the rebound percept is no more common than the other percepts. Both differences are probably attributable to the absence of a transient rebound in the ramp experiment.

Figure 7 brings together the results from the two experiments. Panels A and B compare the peak rebound at ∼12 s (white arrows, Figure 3, N.B. varies somewhat) in Experiment 1 to the rebound at initial equiluminance (15 s) in Experiment 2. The choice of time points to compare is somewhat arbitrary given the differences in the experiments, and the variation is not included because we are not persuaded of the legitimacy of a statistical inference here. However, the qualitative pattern of results across conditions is similar in the two experiments, but with more dominance of the local rebound effect in Experiment 1. (This does not depend on the choice of 15 s as the reference point in Experiment 2: comparing Figures 3 and 5, one can see that the peak rebound effect in Experiment 1 exceeds the maximum of State 2 *at any* time in Experiment 2.) In both experiments, the rebound state is substantially smaller in the coaxial counter-rotation conditions compared with the other conditions.

Figure 7(c) shows that the fraction of corotation is essentially the same across comparable conditions at trial’s end in Experiments 1 and 2. Coaxial conditions have very high corotation at trial’s end with a suggestion of higher corotation in the initially corotating conditions (A > C). This tendency is present in the parallel conditions as well (B > D). This presumably reflects the fact that in A and B, the initial state and its complementary rebound state involve corotation, while in B and D, the initial state and complementary rebound state are opposed by the tendency for corotation.

## Discussion

From several earlier studies, it was known that arrays of objects ambiguously rotating in depth tend to be seen as rotating together. When reduced from many objects to two, this effect is typically weak for parallel kinetic dot objects, but very strong for coaxial ones (Dobbins et al., 1998; Eby et al., 1989; Long & Toppino, 1981). With orthographically projected line segments, Gillam’s group has shown that multiple factors affect rotational grouping including: shared axis of rotation (Gillam & McGrath, 1979), relative line orientation (Gillam, 1972), the fractional (gap to line) separation (Gillam, 1981; Gillam & Grant, 1984), and closure (Gillam, 1975; for a review: Gillam, 2005).

When we set out to do these experiments, we wondered if coaxial rotational coupling might involve a race condition in which the “rotating object” that first perceptually emerges from the set of moving dots captures the rotation of the other. If true, then controlling the initial conditions so that each object is initially unambiguous would obviate the race condition explanation in the sense that the system is placed in a specified state and there is no opportunity for an accidental capture of one object’s rotation by the other as the percept initially emerges. Strikingly, however, coaxial objects remain resistant to being perceived as counter-rotating in our experiments. In neither of the two experiments did it prove possible to substantially increase the likelihood of perceiving coaxial counter-rotation, except transiently in Experiment 1. In that experiment, the rear surface dots suddenly appear, and it is likely that a combination of the transient response to motion onset combined with the adaptation of response in the direction that has been stimulated for 10 s combine to cause the abrupt perceptual reversal.

In a preliminary experiment, observers engaged in a variation of Experiment 1 with a single object in which the prestep portion of the trial had variable length (3, 9, or 27 s), followed by a step transition to transparency. Increasing the initial phase of the trial increased the time spent in the rebound state, consistent with a classical motion aftereffect. However, examination of the data in Figure 2 shows that the dynamics are quantitatively rather different for the different observers. Observer ACD shows only a very brief rebound effect, while observer JOEL’s rebound lasts for the duration of the trial (Figure 2(a)). Differences in latency of the first response and variability of latency (Figure 2(a): observer HONG) also explain why the rebound effect is less than complete when averaged across observers (Figure 3(a)). The other qualitative aspect of observer variability occurs in the coaxial counter-rotation conditions (Figures 2(b) and 3(c)). Recall that here the peak rebound effect is quite small when averaged (38%, Figure 3(c)). Observers showed either a mix of brief rebound effect or an immediate transition to perception of corotation, bypassing the rebound state altogether (Figure 2(b)).

The rapid motion onset of the previously invisible dots, most likely produces a transient response in the neural population sensitive to the direction of the just-appeared dots. This phenomenon probably has a similar basis to the experimental manipulation used in binocular rivalry, in which rapidly switching a binocularly visible grating to a different orientation in only one eye causes the new orientation to be visible at the expense of the previous one. Unlike binocular rivalry, the competition is not for visibility, but for representing the front surface of the object, which determines the rotation direction. In Experiment 1, 10 s of visibility of one direction of motion (and rotation) has the additional effect of adapting low-level direction-selective neurons as well as neurons that represent more sophisticated properties such as surface shape derived from motion. (However, the present experiments do not distinguish between a classical low-level motion aftereffect and higher level effects involving surface convexity or concavity or object rotation.) Therefore, after the initial switch to the rebound percept, we would expect there to be a persistent but declining advantage for the most-recently visible dots in representing the front surface as seen in the slow decline of the fraction of rebound state over the latter part of the trial (Figures 3 and 5).

The results of the second experiment are different from the step experiment in that there is not a transient peak in the rebound effect—just a slow increase in probability of switching to the rebound state that begins within a few seconds of ramp onset. On the other hand, the results are also qualitatively similar to Experiment 1 as can be seen by comparing Figures 3 to 5 and Figures 4 to 6. The distribution of time in each state (Figures 4 and 6) is qualitatively similar, with a greater bias toward the rebound state in the step experiment. This is particularly true in the parallel counter-rotation condition. Figure 7 summarizes a comparison between the two experiments. One reviewer rightly pointed out the danger of choosing a single time point as region of interest for comparison. The point is well-taken. However, the choice of peak rebound is not arbitrary for the step Experiment, although the choice of 15 s as the point in the Ramp experiment certainly is. If one examines Figure 5 closely, it is clear that there is not a sensitive-dependence on the choice of time point in the result—any point in the 14 to 16 s range yields about the same answer. The main point of the summary shown in Figure 7(a) and (b) is that the overall pattern is the same—with the coaxial counter-rotation conditions having much less rebound effect than the other conditions. This is demonstrated more robustly in terms of total time in state (Figures 4 and 6). Finally, Figure 7(c) shows that by the end of trial, the differences associated with the initial condition (A vs. C and B vs. D) have dissipated with only a small residual effect on the amount of corotation observed. One way of thinking about the result shown in Figure 7 is that the dots that ramp up slowly in luminance drive the direction-selective cells less initially (compared with Experiment 1), while the direction-selective neurons driven by the initially visible high luminance dots decrease in response due to adaptation, with the two populations crossing in activity at some point during the ramp, leading to a perceptual switch.

Why are coaxial ambiguous objects so strongly inclined to rotate together? One kind of answer is provided by Occam’s razor—corotation is the simplest explanation of the data. On the other hand, is this not the simplest explanation of the data in the parallel axis case as well? We can think of the setup in our experiments in at least two different ways. In one, object motion is to be explained as much as possible (most generically) in terms of observer motion. In another, motion is attributable to the objects and not the observer. Of course, we can also imagine a combination of the two. From a theorem of mechanics known as Chasles’ Theorem (Chasles, 1830) and as clearly expressed by Whittaker (1904): “A rotation about any axis is equivalent to a rotation through the same angle about any axis parallel to it, together with a simple translation in a direction perpendicular to the axis.”

**Figure 8.**
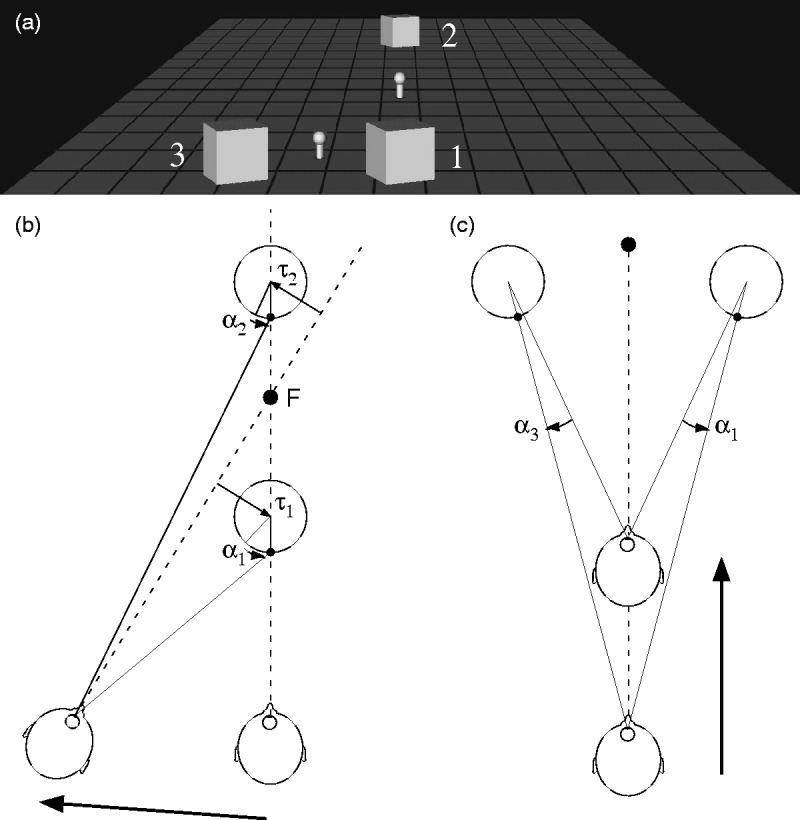
Object translation and rotation for a moving observer. (a) Three cubes and fixation objects. The drawings in the next two parts of the figure consider Objects 1 and 2 (b) or 1 and 3 (c) under different movement regimes. (b) Moving to the left while fixating on the central object yields a combination of translation (τ) and rotation (α) in Objects 1 and 2. Translation depends on the eye movement during observer movement, but the object rotation does not. (c) Moving forward between Objects 1 and 3 leads to opposite rotation in the two objects. (N.B. Object translation is not depicted in this case, nor is object expansion).

In the present context (Figure 8), this can be interpreted to mean that a pure translation of the observer can be equally conceived as a combination of rotation and translation of the objects. Consideration is narrowed to the case where an eye movement is employed to render stationary on the retina a particular fixation point (F). In the transverse parallax scenario (Figure 8(b)), two initially aligned objects both translate (τ) and rotate (α) on the retina. Object translation direction and speed depends on the rotation of the eye (which point in the scene is fixated) in addition to distance. In contrast, object rotation does not depend on the eye rotation. Therefore, there are clear benefits for the visual system to decompose object motion into translational and rotational components.

With transverse parallax (Figure 8(b)), initially aligned objects (1 and 2) rapidly become misaligned and rotate at different rates because they are at different distances. In contrast, if Object 2 were directly above Object 1 (coaxial), their translation and rotation would be identical. In other words, in the ego motion regime, apparently coaxial objects with identical motion can be explained by transverse parallax. As an example, think of walking past a tree trunk that is partially occluded so that it forms two cylinders. Analogously with the present experiments, the two parts of the tree trunk share common translation and rotation. In Figure 8(c), motion along a trajectory between two objects generates opposite rotation and translation (as well as expansion). The important thing here is that in the parallel configuration in our experiments, same-spin and opposite-spin can both be accounted for by particular ego motions, but there is no ego motion that can generate coaxial counter-rotation.

Although ego motion-generated visual motion probably represents the overwhelming majority of our experience, we also commonly experience multiple independently moving objects, and we are capable of generating hypotheses to account for these situations. Indeed, as pointed out in Figure 1(d; and Demo 2), with increasing tilt and with common spin pitted against common axis, we become increasingly willing to opt for a common axis interpretation as tilt increases (see also Dobbins & Grossmann, 2010; Grossmann & Dobbins, 2006). At higher tilt, shared object symmetry prevails over spin direction. Occam’s razor counsels that we favor simple explanations over complex ones when both account equally well for the data. MacKay (1991, 2003) goes one better: “Coherent inference (as embodied by Bayesian probability) automatically embodies Occam’s razor, quantitatively.”

His point is that a simple model in covering less of the data space is more probable or predictive than a more complex model that spreads its predictive power over more of the data space (see MacKay, 2003, for examples). In the current context, the simplest model is the one that attempts to account for the data in terms of ego motion, while a more complex model permits independent object motion or combines ego motion with object motion. A natural objection is that the participant in these experiments has no evidence of undergoing ego motion—she is sitting in a chair watching what appear to be stationary spinning objects. Nevertheless, if the form-from-motion apparatus operates on the instantaneous motion fields rather than integrating over time, the constraints derived from ego motion analysis may well prevail.

Finally, it is worthwhile to return to the point that the viewing condition in our experiments degenerate in a certain sense—axis and spin sense correspond—axial alignment and common spin are in agreement. Corotation is considered as perceptual grouping and opposite rotation as not (or “fragmentation” in the terminology of Gillam). Yet, under a variety of conditions, the fragmented percept can also be an example of perceptual grouping. For instance, in Experiment 1 of Gillam (1972), alternative perceptual interpretations are possible, and possibly with different likelihoods as a function of the degree of divergence of the two lines. One of those interpretations is akin to Demo 2 in which tilted, rotating cylinders can be seen to have opposite spin with a shared axis or common spin with oppositely tilted axes. This case in which spin and axis are decoupled represents a kind of symmetry-breaking not present in the standard orthogonally viewed display, and shows that one can invoke different models or hypotheses to explain the data. The common spin model is compatible with the ego-translation hypothesis, while the common axis model requires the assumption of independent, spinning objects.

## Supplementary Material

Supplementary material

Supplementary Movie 1

Supplementary Movie 2

Supplementary Movie 3

Supplementary Movie 4

Supplementary Movie 5

Supplementary Movie 6

Supplementary Movie 7

Supplementary Movie 8

Demo 1

Demo 2

## Author note

Author Jon K. Grossmann is affiliated to SoundHound, Inc. 979 Freedom Cir., Suite 400, Santa Clara, CA 95054, USA.
