# Supplementary material for: Can Rotational Grouping Be Determined by the Initial Conditions?
Source: Iperception. 2018 Jan 22;9(1):2041669517748338. doi: 10.1177/2041669517748338 (PMC5784469; doi:10.1177/2041669517748338)
Supplement: Supplementary material [file supplementary_material.pdf]

Table 1. Guide to Movie Files.

		Movie file
Demo 1	Figure 1(c)	Coax-cylinders-rotating-bkgd
Demo 2	Figure 1(d)	Tilted-Coaxial-Cylinders
Experiment 1	Figure 3(a)	InstantTransparency_Coax_CoRotation
	Figure 3(b)	InstantTransparency_Parax_CoRotation
	Figure 3(c)	InstantTransparency_Coax_Counter
	Figure 3(d)	InstantTransparency_Parax_Counter
Experiment 2	Figure 5(a)	GradualTransparency_Coax_CoRotation
	Figure 5(b)	GradualTransparency_Parax_CoRotation
	Figure 5(c)	GradualTransparency_Coax_Counter
	Figure 5(d)	GradualTransparency_Parax_Counter

Video Legends

N.B. It is possible to rescale the videos as desired by changing the window size. Also, depending on the version of QuickTime Player used, it may be necessary to move the mouse off the window to eliminate the controls (which can obscure the display).

Demo 1. (Coax-cylinders-rotating-bkgd.mov) Ambiguous rotation rendered unambiguous by moving background. Coaxial cylinders—ordinarily seen as corotating are biased oppositely by rotating background.

Demo 2. (Tilted-Coaxial-Cylinders) A pair of cylinders with axes tilted 10° out of the screen. Observers have a choice between seeing them have a shared tilt axis and opposite spin direction, or opposite axial tilt and shared spin. At this degree of tilt, observers switch between the two interpretations. At higher tilts, common axis—opposite spin dominates.

Experiment 1

Video 1. (InstantTransparency_Coax_CoRotation.mov) Figures 2(a) and 3(a)

Video 2. (InstantTransparency_Parax_CoRotation.mov) Figure 3(b)

Video 3. (InstantTransparency_Coax_Counter.mov) Figures 2(b) and 3(c)

Video 4. (InstantTransparency_Parax_Counter.mov) Figure 3(d)

Experiment 2

Video 5. (GradualTransparency_Coax_CoRotation.mov) Figure 5(a)

Video 6. (GradualTransparency_Parax_CoRotation.mov) Figure 5(b)

Video 7. (GradualTransparency_Coax_Counter.mov) Figure 5(c)

Video 8. (GradualTransparency_Parax_Counter.mov) Figure 5(d)
